# Deconstructing cellular senescence in bone and beyond

**DOI:** 10.1172/JCI169069

**Published:** 2023-04-17

**Authors:** Lorenz C. Hofbauer, Franziska Lademann, Martina Rauner

**Affiliations:** 1 Division of Endocrinology, Diabetes and Bone Diseases, Department of Medicine III and University Center for Healthy Aging and; 2 Center for Regenerative Therapies Dresden, Technische Universität Dresden, Dresden, Germany.

## Abstract

Osteocytes are specialized bone cells that orchestrate skeletal remodeling. Senescent osteocytes are characterized by an activation of cyclin-dependent kinase inhibitor p16^Ink4a^ and have been implicated in the pathogenesis of several bone loss disorders. In this issue of the *JCI*, Farr et al. have now shown that systemic removal of senescent cells (termed senolysis) prevented age-related bone loss at the spine and femur and mitigated bone marrow adiposity through a robust effect on osteoblasts and osteoclasts, whereas cell-specific senolysis in osteocytes alone was only partially effective. Surprisingly, transplantation of senescent fibroblasts into the peritoneum of young mice caused host osteocyte senescence associated with bone loss. This refined concept of osteocyte senescence and the effects of remote senolysis may help to develop improved senolytic strategies against multisystem aging in bone and beyond.

## Bone health and the osteocyte

Maintaining lifelong mobility is one aim of healthy aging that allows independence and autonomy. However, falls and fragility fractures, which tend to occur in clusters toward the end of life, represent common hazards for the mobility of the aging population. This period comes with a substantial loss of quality of life and causes an enormous socioeconomic burden for patients and their families (1). While there has been tremendous progress in our understanding of osteoporosis due to sex hormone deficiency or medications, insights into how cell-intrinsic mechanisms contribute to the aging process of the skeletal system are still limited. Similar limitations apply to the availability of specific measures that help to prevent or reverse skeletal aging. Moreover, the cellular abnormalities that connect muscle, bone, and fat tissue en route to sarcopenia, osteoporosis, and obesity have remained elusive. As a result, many older patients with osteoporosis receive rather generic clinical recommendations, such as to increase physical activity, improve lifestyle and diet, and use antiresorptive drugs (2), all of which have established benefits but are not bona fide targeted therapies.

Over the past decade, emerging bone research has focused on the biology of osteocytes, the least accessible yet most common bone-resident cell type (3). Osteocytes are mechanosensing stellate cells embedded in the bone mineral that communicate via a lacunocanalicular network, giving them a neuronal network appearance. By producing key signaling molecules of the Wnt signaling and RANKL pathways, osteocytes control osteoblastic bone formation and osteoclastic bone resorption and govern bone remodeling — the coupling of these two processes (3). Earlier studies have indicated that increased osteocyte apoptosis contributes to osteoporosis following glucocorticoid exposure in mice and humans (4).

## Senescent cells in age-related bone loss disorders

**Figure 1:**
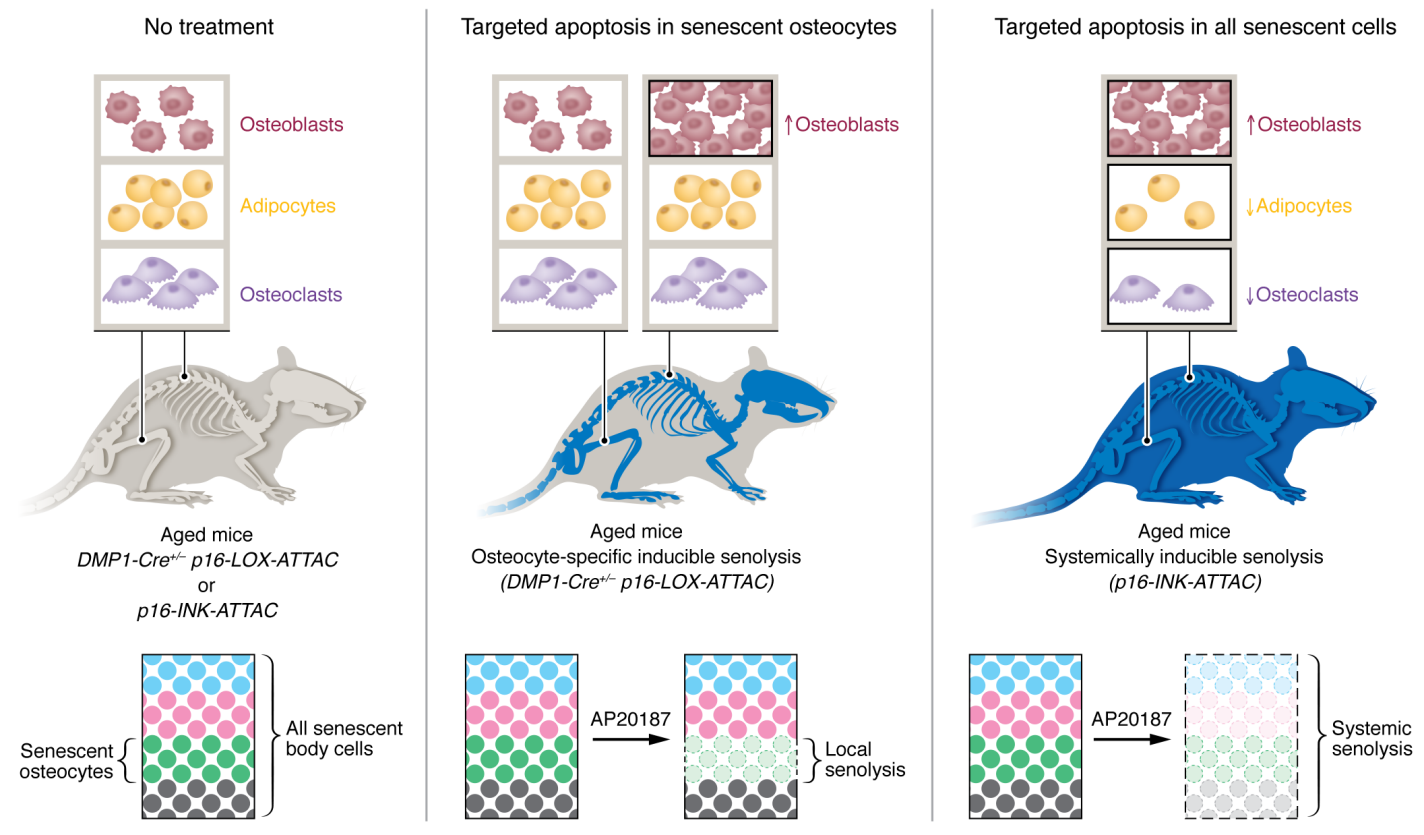
Beneficial effects of osteocyte-specific versus systemically inducible senolysis on bone remodeling in aged mice. In aged *DMP1-Cre^+/–^ p16-LOX-ATTAC* mice, DMP promoter–driven elimination of only senescent osteocytes upon administration of a synthetic drug (AP20187) preserves vertebral, but not femoral, bone mass by improving bone formation without affecting osteoclast or bone marrow adipocyte numbers. Systemically induced removal of all senescent cells with AP20187 in *p16-INK-ATTAC* mice hampered age-driven bone loss and led to improved bone formation, suppressed bone resorption, and reduced bone marrow adiposity.

The increased appearance and persistence of senescent cells is a hallmark of cellular aging (5). Senescent cells are characterized by an activation of the cyclin-dependent kinase inhibitors p16^Ink4a^ and p21^Cip1^ in response to stress, resulting in DNA double-stranded breaks at telomeres (telomere-associated foci [TAF]) and the secretion of a senescence-associated secretory phenotype (SASP). This SASP, a biochemically heterogeneous mixture of factors, promotes chronic inflammation and alters the cellular function of surrounding organs. Senescent cells have been receiving increasing attention from both basic science and translational perspectives because they can be eliminated by senolytic drugs, which may slow down the aging process. In a coherent series of studies by Mayo Clinic researchers, senescent osteocytes have been implicated across a broad spectrum of bone loss disorders, including age-related bone loss (6), diabetic bone disease (7), periodontal infection–associated bone loss (8), and focal bone loss following radiotherapy (9). Moreover, Farr and colleagues demonstrated that senolytic strategies that (a) genetically eliminate p16^Ink4a^-positive senescent cells, using *p16-INK-ATTAC* (apoptosis through targeted activation of caspase 8) — an inducible suicide transgene driven by the *p16^Ink4a^* promoter, or (b) pharmacologically suppress the production of the SASP secretome using a JAK inhibitor, prevented both age-related bone loss and deterioration of bone remodeling and strength (6). However, it was still unclear whether elimination of senescent cells locally in bone, or systemically in different systems accounted for these beneficial senolytic effects. In this issue of the *JCI*, Farr et al. have now provided decisive answers (10). To discern between local and systemic effects, they developed another mouse model using the *p16-LOX-ATTAC* transgene with the *DMP* promoter to induce senolysis only in senescent osteocytes (termed “local senolysis”) upon administration of a synthetic drug (AP20187). The authors used stringent criteria, TAF staining and expression of *p16^Ink4a^*, to document senescence at a single-cell level. The results were compared with those of the established *p16-INK-ATTAC* mouse model, targeting all senescent cells (termed “systemic senolysis”) (6). Striking differences were noted. While elimination of senescent osteocytes prevented age-related spinal bone loss, it had no effect on the femur. In addition, local senolysis in bone improved bone formation, but did not affect osteoclastic bone resorption or bone marrow adipocytes. By contrast, systemic senolysis helped to preserve bone mass at the spine and femur through a dual effect with (a) improved bone formation and suppressed bone resorption and (b) a reduction of bone marrow adiposity (Figure 1). These beneficial systemic senolytic effects were consistent for cortical and trabecular bone and were similar for aged male and female mice despite some minor differences. As the authors acknowledge, minimal differences in the transgene construct between the systemic (*p16-INK-ATTAC*) and the local/cell-specific (*DMP1-Cre^+/–^* *p16-LOX-ATTAC*) mouse models may represent a potential confounder. Furthermore, DMP1-Cre promoter–driven knockout might also target late osteoblasts, which may have driven the bone changes seen in *DMP1-Cre^+/–^* *p16-LOX-ATTAC* mice as well. Since a small fraction of osteocytes become senescent with aging (10% or less), and senolysis in *DMP1-Cre^+/–^* *p16-LOX-ATTAC* mice removes only 30% of the senescent osteocytes, the possibility remains that targeting more senescent osteocytes could achieve a greater effect.

In any case, it is remarkable that senolysis of a small fraction of senescent osteocytes may account for the robust bone-protective effect, further supporting the importance of osteocytes for bone metabolism and skeletal health. Given their crucial role in orchestrating bone remodeling and their high degree of connection, these few senescent osteocytes may be sufficient to cause functional chaos through cellular miscommunication.

Finally, the authors demonstrated that transplantation of senescent nonskeletal fibroblasts generated by irradiation into the peritoneal cavity of young mice caused skeletal aging with senescence within host osteocytes and bone loss. These findings are intriguing and corroborate the concept of systemic senescence related to skeletal aging or what they termed “senescence at a distance” (10), emphasizing the need for systemic senolytics to maintain bone health in old age.

## A systems biology approach in the quest for senolytics

Diabetic bone disease shares some features with accelerated skeletal aging, including cortical porosity, disturbed lacunocanalicular networks, and enhanced osteocyte senescence that translate into reduced bone strength (7, 11). It has been hypothesized that cortical porosity, Swiss cheese–like structure defects of the outer bone shell, in type 2 diabetes mellitus could be linked to vascular or immune effects (11). In fact, the study by Farr et al. does not rule out the contribution of other cell types after systemic senolysis to reduce bone resorption (10). These other cell types may include vascular endothelial cells and activated T cells, which produce the osteoclast differentiation factor RANKL. Notably, RANKL is reduced by systemic senolysis. Since the Farr et al. study reported a reduction in sclerostin, an important inhibitor of Wnt signaling and osteoblastic bone formation in local and systemic senolysis (10), it would be interesting in future studies to assess whether, vice versa, bone-anabolic therapies (teriparatide, abaloparatide, or the sclerostin antibody romosozumab) may interfere with osteocyte senescence or confer senolytic properties.

As shown by the Farr et al. study, senescence in multiple cell types may need to be targeted to achieve robust effects on the skeleton (10). Thus, also at an organismal level, approaches targeting several systems should be considered. Recent randomized, controlled clinical trials on healthy aging have assessed several interventions, such as vitamin D, omega-3 fatty acids, and structured exercise in a broad array of relevant age-related outcomes (12, 13). For example, the US-based VITAL study assessed the effects of vitamin D and marine omega-3 fatty acid supplements on the primary prevention of cardiovascular disease and cancer (12). Moreover, the European DO-HEALTH study evaluated the effects of vitamin D supplements, omega-3 fatty acid supplements, and a defined strength-training exercise program on falls, fractures, cognitive function, infections, and blood pressure (13). A similar systems biology approach is warranted for the future evaluation of senolytic drugs across the continuum of age-related diseases, including musculoskeletal, cardiovascular, and neurodegenerative disorders.

## Version 1 04/17/2023

Electronic publication
